# miR‐1‐3p and miR‐206 sensitizes HGF‐induced gefitinib‐resistant human lung cancer cells through inhibition of c‐Met signalling and EMT

**DOI:** 10.1111/jcmm.13629

**Published:** 2018-04-17

**Authors:** Demin Jiao, Jun Chen, Yu Li, Xiali Tang, Jian Wang, Wei Xu, Jia Song, You Li, Huimin Tao, Qingyong Chen

**Affiliations:** ^1^ Department of Respiratory Disease The 117th Hospital of PLA Hangzhou Zhejiang China; ^2^ The First Affiliated Hospital of Wenzhou Medical University Wenzhou Zhejiang China

**Keywords:** c‐Met, EMT, gefitinib resistance, lung cancer, miR‐1‐3p, miR‐206

## Abstract

Hepatocyte growth factor (HGF) overexpression is an important mechanism in acquired epidermal growth factor receptor (EGFR) kinase inhibitor gefitinib resistance in lung cancers with EGFR activating mutations. MiR‐1‐3p and miR‐206 act as suppressors in lung cancer proliferation and metastasis. However, whether miR‐1‐3p and miR‐206 can overcome HGF‐induced gefitinib resistance in EGFR mutant lung cancer is not clear. In this study, we showed that miR‐1‐3p and miR‐206 restored the sensitivities of lung cancer cells PC‐9 and HCC‐827 to gefitinib in present of HGF. For the mechanisms, we demonstrated that both miR‐1‐3p and miR‐206 directly target HGF receptor c‐Met in lung cancer. Knockdown of c‐Met mimicked the effects of miR‐1‐3p and miR‐206 transfections Meanwhile, c‐Met overexpression attenuated the effects of miR‐1‐3p and miR‐206 in HGF‐induced gefitinib resistance of lung cancers. Furthermore, we showed that miR‐1‐3p and miR‐206 inhibited c‐Met downstream Akt and Erk pathway and blocked HGF‐induced epithelial‐mesenchymal transition (EMT). Finally, we demonstrated that miR‐1‐3p and miR‐206 can increase gefitinib sensitivity in xenograft mouse models *in vivo*. Our study for the first time indicated the new function of miR‐1‐3p and miR‐206 in overcoming HGF‐induced gefitinib resistance in EGFR mutant lung cancer cell.

## 1 INTRODUCTION

Lung cancer is one of the leading causes of cancer‐related death worldwide. Targeted therapy with tyrosine‐kinase inhibitors (TKI) including gefitinib has dramatically improved the rates of response and survival in advanced epidermal growth factor receptor (EGFR)‐mutated non‐small cell lung cancer (NSCLC). Unfortunately, drug resistance will eventually develop in the majority of the patients. The secondary EGFR T790M mutation accounts for about half of acquired resistance mechanisms.^1^ As a bypass pathway, c‐Met activation caused by c‐Met gene amplification, mutation and/or its ligand hepatocyte growth factor (HGF) overexpression has been reported as another important mechanism in this process.^2^ In particular, HGF overexpression was observed in about 61% of patients with acquired resistance.^3^ Therefore, targeting HGF/c‐Met pathway is important for more effective targeted therapy in lung cancer.

MicroRNAs (miRNAs) are endogenously expressed small non‐coding RNA (about 22‐25 nucleotides in length) acting as post‐transcriptional regulators of gene expression. MiRNA can influence many biological processes, such as cell proliferation, invasion, metastasis, differentiation, metabolism and apoptosis. Multiple miRNAs have been reported down‐regulated in lung cancer and acted as suppressors in tumour development, including miR‐145,^4^ let 7,^5^ miR‐1,^6^ miR‐34b/c,^7^ miR‐449a,^8^ and so on. miRNA‐1 (mainly refer to miR‐1‐3p) and miR‐206 are members of the muscle‐specific miR‐1 family of so‐called myomiRs,^9^ which play an important role in the myogenesis and development of cardiac and skeletal muscles.^10^, ^11^ A multitude of recent studies showed that deregulation of myomiR expression is associated with a variety of cancers.^12^, ^13^, ^14^, ^15^ In lung cancer, miR‐1 and miR‐206 have been found exhibited antitumour activities in multiple aspects, including repression of cell proliferation, migration, invasion and angiogenesis. For example, Korde et al^12^ found that miR‐1 levels were lower in NSCLC than cancer‐free tissue and overexpression of miR‐1 could reduce tumour growth and angiogenesis by targeting Mpl gene. Chiu et al^16^ showed that ADAM9 down‐regulated miR‐1 expression, which in turn enhanced CDCP1 expression to promote lung cancer progression. Singh et al^17^ reported that down‐regulation of miR−1/−206 in NSCLC has major effects on carbon flux and the activity of metabolic pathways associated with cell proliferation and growth. Furthermore, we and other researchers have found that low expression of miR‐206 is related to lung cancer invasion and metastasis.^18^, ^19^ However, the role of miR‐1‐3p and miR‐206 in HGF‐induced gefitinib resistance of lung cancer is not clear.

This study was performed to investigate whether miR‐1‐3p and miR‐206 increased the sensitivity of HGF‐induced gefitinib resistance in EGFR mutant lung cancer cells. We assessed this issue using human lung cancer cells, PC9 and HCC‐827, both harbouring deletions in exon19 of EGFR. The results showed that HGF‐induced gefitinib resistance and miR‐1‐3p and miR‐206 could circumvent the HGF‐induced gefitinib resistance by targeting c‐Met‐Akt‐Erk pathway and suppressing epithelial‐mesenchymal transition (EMT).

## 2 MATERIALS AND METHODS

### 2.1 Cell cultures

The EGFR mutant (exon 19 deletion) human lung adenocarcinoma cell lines, PC‐9, were provided by Affiliated Hangzhou Hospital of Nanjing Medical University. HCC827 cell line was purchased from the Cell Bank at the Chinese Academy of Sciences. Both the cell lines were maintained in RPMI 1640 medium supplemented with 10% FBS and antibiotics.

### 2.2 miRNA transfection

The miRNA mimics or negative control mimics were chemically synthesized by GenePharma Co., Ltd. (shanghai, China), and the transfection was conducted as we described previously.^20^ All miRNA sequences are listed in Table S1. Transfection efficiency was confirmed by SYBR green (Takara) real‐time PCR detection of miR‐1‐3p and miR‐206 expression.

### 2.3 c‐Met knockdown and overexpression

For knockdown of c‐Met, c‐Met shRNA expression vector (PGPU6/GFP/neo‐shRNA‐c‐Met, designated as sh‐Met) and the control vector PGPU6/GFP/neo‐shControl (designated as sh‐NC) were provided by GenePharma Inc (Shanghai, China). c‐Met shRNA sequences are listed in Table S2. For overexpression of c‐Met (Accession NO.: NM_000245), c‐Met overexpression vector (pEZ/M98/neo‐c‐Met, designated as ex‐Met) and the control vector (pEZ/M98/neo‐control, designated as ex‐NC) were provided by GeneCopoeia Inc (Guangzhou, China). All the vectors were transfected by Lipofectamine 2000 (Invitrogen) reagent according to the manufacturer's instructions. Transfection efficiency was confirmed by Western blotting detection of c‐Met expression.

### 2.4 HGF overexpression

Hepatocyte growth factor overexpression lentivirus (Leti‐Ubi‐MCS‐3FLAG‐SV40‐puromycin‐HGF) was constructed by GeneCHEM inc (Shanghai, China). PC‐9 and HCC827 cells were infected in accordance with the manufacturer's instructions. After puromycin selection, HGF‐producing cell lines, PC‐9/HGF and HCC827/HGF, were established. HGF production in these cells was determined by ELISA method.

### 2.5 Cell viability assay

The cell viability was detected by MTT method. Tumour cells (4 × 10^3^ per well) were plated into 96‐well plates and allowed to adhere overnight. Then, the cells were transfected with miRNA mimics or mimic NC. After 24 hours of incubation, different concentrations of gefitinib (0.001‐1 μmol/L) and/or HGF were added, and incubation was continued for 48‐72 hours. The cell survival rate was determined with MTT solution (5 mg/mL; Sigma). Each sample was plated in quintuplicate, and three independent experiments were performed.

### 2.6 Migration and invasion assay

Wound healing experiment and transwell assays were used to determine the migration and invasion abilities of the cells, respectively. The experiments were conducted as we described previously.^21^

### 2.7 Quantitative RT‐PCR

Total RNA was isolated from tumour cells by Trizol reagent (Invitrogen) following manufacture's protocol. cDNA was synthesized by cDNA Synthesis Kit (Invitrogen), and RT‐PCR was performed using SYBR green methods (Takara). U6 RNA was used as a miRNA internal control. The primers for miR‐1‐3p and miR‐206 were in Table S3.

### 2.8 Western blot analysis

Cells were harvested and lysed on ice for 30 minutes in RIPA buffer (Beyotime) supplemented with protease inhibitor cocktail (Roche). Lysates were subjected to Western blotting assay as described previously^21^ and detected with antibodies against p‐Met, c‐Met, p‐EGFR, EGFR, phospho‐AKT, total AKT, phospho‐ERK, total ERK, GAPDH, E‐cadherin, Vimentin and Snail (Cell signalling technology).

### 2.9 Immunofluorescence staining

Cells cultured on glass bottom culture dish were transfected with miRNA mimics or their mimic control for 24 hours and then fixed with 4% formaldehyde for 10 minutes and permeabilized with 0.1% Triton X‐100. Fixed cells were incubated in blocking buffer (PBS, pH 7.4, 0.1% Triton X‐100 and 1% bovine serum albumin) and stained with primary antibodies for vimentin (Cell signailling technology), E‐cadherin (Cell signailling technology) over night and then with secondary antibodies labelled with TRITC (Invitrogen) for 1 hour at room temperature. Nuclei were stained for 3 minutes with Hochest 33342 (1 mg/mL). Confocal analysis was performed on a NIKON C2 confocal microscope, and the images were taken under the same parameter configuration.

### 2.10 Luciferase reporter assay

Luciferase reporter constructs containing portions of the c‐Met 3′‐UTR (pmirGLO‐c‐Met‐wt), mutant sequence (pmirGLO‐c‐Met‐mut) were generated by GenePharma Inc (Shanghai, China). The inserted sequence are shown in Table S4; Cells were co‐transfected with 25 ng of c‐Met 3′‐UTR reporter constructs and 20 nmol/L miR‐1‐3p or miR‐206 mimics using Lipofectamine 2000 (Invitrogen) in 24‐well plates. Luciferase assays were performed 24 hours after transfection using the Dual‐Luciferase reporter assay system (Promega). Firefly luciferase activity was normalized to Renilla luciferase activity.

### 2.11 Animal studies

All experimental procedures involving animals in this study had been approved by the ethics committee in the 117th Hospital of PLA. Male nude mice (BALB/c, 4 weeks old) were purchased from Shanghai Laboratory Animal Center (Shanghai, China). For preparation of subcutaneous xenograft model, PC‐9/HGF cells (10^7^ cells in 0.2 mL physiological saline per mouse) were injected subcutaneously into the rear flank of the nine nude mice. PC‐9/NC cells were also injected one mouse to as a positive control (gefitinib‐sensitive group). Ten days after tumour cell inoculation with confirmation of successful maturation of tumours, 9 mice injected with PC‐9/HGF cells were divided randomly into three groups (GE+agomir NC group, GE+miR‐1‐3p agomir group and GE+miR‐206 agomir group). Gefitinib (25 mg/kg/d) was used by oral gavage. MiR‐1‐3p and miR‐206 agomirs and their negative control (NC) (2 nmol; Genepharma, Shanghai, China) were given locally by direct injection into the xenografts every 3 days. In addition, we set 3 days of stop gefitinib (stop‐GE) interval to confirm the effect of gefitinib and combined treatment. Tumour volumes were determined (in cubic millimetre) by measuring in two directions and were calculated as tumour volume = length × (width)^2^/2.

### 2.12 Statistical analysis

All data were presented as the means ± standard deviation. Differences between samples were analysed using two‐tailed Student's *t* test. *P* < .05 was considered statistically significant.

## 3 RESULTS

### 3.1 HGF induced gefitinib resistance and decreased expression of miR‐1‐3p and miR‐206 in EGFR mutant lung cancer cells

**Figure 1:**
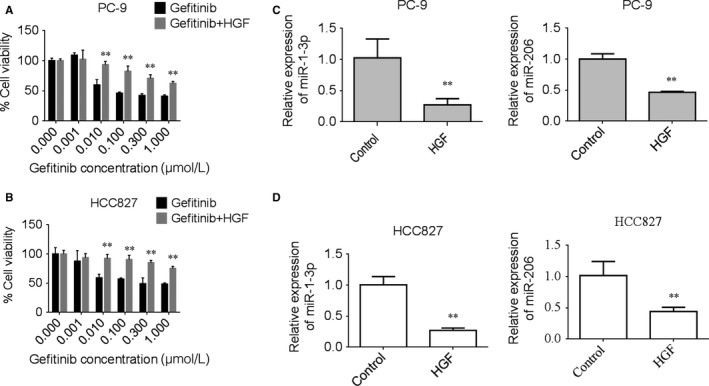
HGF induced gefitinib resistance and down‐regulated expression of miR‐1‐3p and miR‐206 in lung adenocarcinoma PC‐9 and HCC827 cells. A‐B, HGF induced gefitinib resistance in PC‐9 (A) and HCC827 (B) cells. Tumour cells were incubated with increasing concentrations of gefitinib in the presence/absence of HGF, and cell viability was determined after 72 h of treatment by MTT assay. Data are means of three separated experiments ± SD, **P <* *.01 compared with gefitinib group. C‐D, HGF‐induced down‐regulated expression of miR‐1‐3p and miR‐206 in PC‐9 (C) and HCC827 cells (D). Tumour cells were incubated with HGF (50 ng/mL) for 72 h, the expression of miR‐1‐3p and miR‐206 were determined by QPCR assay. Data are means of three separated experiments ± SD, **P <* *.01 compared with control group

PC‐9 and HCC827 cell lines (both harbouring EGFR exon 19 deletion) are highly sensitive to gefitinib. Our MTT results showed that HGF can induce gefitinib resistance in these two cells (Figure 1A,B), which is consistent with previous report.^22^ In addition, we found that HGF slightly promoted proliferation of PC‐9 and HCC827 cells under our experimental conditions (Figure S1A,B), but significantly enhanced the migration and invasion activities of two cells (Figure S1C‐F).

miR‐1‐3p and miR‐206 have been identified as tumour suppressors in various human cancers, including lung cancer.^6^, ^12^, ^17^, ^18^, ^23^, ^24^ Exogenous expression of these two miRNAs could significantly reduce cell motility and invasiveness.^25^ In this study, we evaluated miR‐1‐3p and miR‐206 expression in HGF stimulation of PC‐9 and HCC827 cells. Real‐time PCR results showed that the expression levels of both miR‐1‐3p and miR‐206 were dramatically decreased as compared with control cells (Figure 1C,D). In addition, we found that gefitinib increased the expression of miR‐1‐3p and miR‐206, whereas HGF attenuated this effect in both cells (Figure S2A,B).

### 3.2 miR‐1‐3p/miR‐206 restores gefitinib sensitivity following treatment with HGF in EGFR mutant NSCLC cell lines

**Figure 2:**
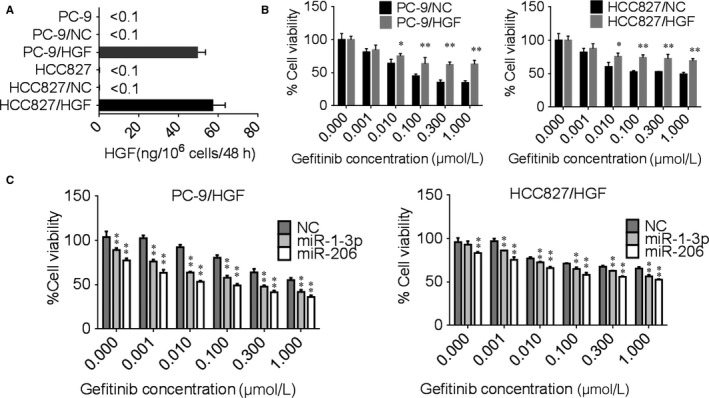
miR‐1‐3p and miR‐206 overcame HGF‐induced gefitinib resistance in PC‐9 and HCC827 cells. A, HGF overexpression lentivirus increased the production of HGF in PC‐9 and HCC827 cells. The cells were incubated in medium contained lentivirus for 48 h, and culture supernatants were harvested. The level of HGF was determined by ELISA. B, HGF overexpressed PC‐9 and HCC827cells increased gefitinib resistance. The PC‐9/NC, HCC827/NC, PC‐9/HGF, HCC827/HGF cells were incubated with increasing concentrations of gefitinib for 72 h. Cell viability was measured by MTT assay. Data are means of three separated experiments ± SD, *P <* *.05, **P <* *.01 compared with control group. C, miR‐1‐3p and miR‐206 mimics transfection reversed HGF‐induced gefitinib resistance. The PC‐9/HGF, HCC827/HGF cells were transfected with miR‐1‐3p or miR‐206 mimics for 24 h and then treated with increasing concentrations of gefitinib. Cell viability was measured by MTT assay. Data are means of three separated experiments ± SD, *P <* *.05, **P <* *.01 compared with negative control (NC) group. PC‐9/NC and HCC827/NC cells: negative control lentivirus infected cells; PC‐9/HGF and HCC827/HGF cells: HGF overexpressed lentivirus‐infected cells

In order to test the effect of high miR‐1‐3p/miR‐206 expression on modulation of gefitinib sensitivity, we constructed stable HGF overexpressed PC‐9 cells (PC‐9/HGF) and HCC‐827 cells (HCC‐827/HGF). ELISA showed that both PC‐9/HGF and HCC‐827/HGF cells secreted high concentrations of HGF (>50 ng/10^6^ cells/48 h). However, HGF secreted by control vector‐transfected PC‐9 and HCC827 cells (PC‐9/NC and HCC‐827/NC) were below the detected limit (Figure 2A). MTT results showed that both PC‐9/HGF and HCC827/HGF cells became resistant to gefitinib than their parent controls (PC‐9 and HCC827 cells) (Figure 2B).

Then, we transfected miR‐1‐3p and miR‐206 mimics to increase the expression of miR‐1‐3p and miR‐206 in PC‐9/HGF and HCC827/HGF cells, MTT assay was used to detect gefitinib sensitivity of these cells. The results showed that either miR‐1‐3p or miR‐206 mimics could obviously reversed HGF‐induced gefitinib resistance compared with mimics negative control (NC) (Figure 2C). These results suggested that miR‐1‐3p/miR‐206 are potential suppressors of HGF‐induced gefitinib resistance in PC‐9 and HCC827 cells.

### 3.3 miR‐1‐3p/miR‐206 target c‐Met in EGFR mutant NSCLC cell lines

**Figure 3:**
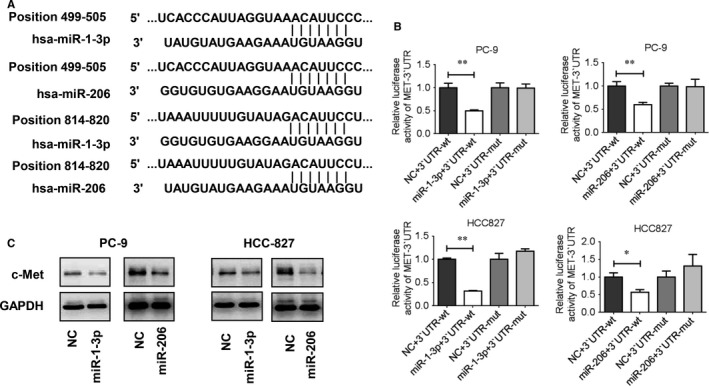
c‐Met is a direct target of miR‐1‐3p and miR‐206 in PC‐9 and HCC827 cells. A, Alignment between the predicted binding motifs within the c‐Met 3′UTRs and miR‐1‐3p/miR‐206. Complementary sequences to the seed regions of miR‐1‐3p/miR‐206 are also indicated. B, Target verification for miR‐1‐3p/miR‐206 in PC‐9 and HCC827 cells. PC‐9 and HCC827 cells were co‐transfected with miR‐1‐3p or miR‐206, pmirGLO‐c‐Met‐wt (3′UTR‐wt) or pmirGLO‐c‐Met‐mut (3′ UTR‐mut), along with a pRL‐SV40 reporter plasmid. After 24 h, the luciferase activity was measured. Values are presented as relative luciferase activity after normalization to *Renilla* luciferase activity. *P <* *.05, **P <* *.01. C, c‐Met expression after miR‐1‐3p or miR‐206 mimics transfection was determined by Western blot analysis in PC‐9 and HCC827 cells

Hepatocyte growth factor and its receptor c‐Met are a ligand‐receptor pair with important functions in various biological processes. In previous study, we have demonstrated that c‐Met is a direct target of miR‐206 in lung cancer 95D and 95C cells.^18^ miR‐1‐3p and miR‐206 belong to the same cluster of miRNAs that are different from each other by only 3 nucleotides. Therefore, they have many predicted and validated common targets, such as ARPC3, c‐Met, VEGFA.^26^, ^27^, ^28^ Figure 3A shows the regions of 3′UTR of the c‐Met gene that harbours 2 conserved miR‐1‐3p and miR‐206 sites based on the prediction of Targetscan software. Here, we further performed luciferase reporter assay to verify that miR‐1‐3p and miR‐206 directly targets c‐Met in PC‐9 and HCC827 cells. The results showed that both miR‐1‐3p and miR‐206 mimics led to a significant decrease in the luciferase activity in c‐Met wild‐type (wt) constructs group, In contract, there is no decrease in mutant type (mut) group (Figure 3B). Western blot analyses showed that the miR‐1‐3p/miR‐206 mimic treatments induced down‐regulation of protein expression of c‐Met in two lung cancer cells (Figure 3C). These data showed that c‐Met is a direct target of miR‐1‐3p/miR‐206 in HCC827 and PC‐9 cells.

### 3.4 miR‐1‐3p/miR‐206 target c‐Met expression plays an important role in HGF‐induced gefitinib resistance

**Figure 4:**
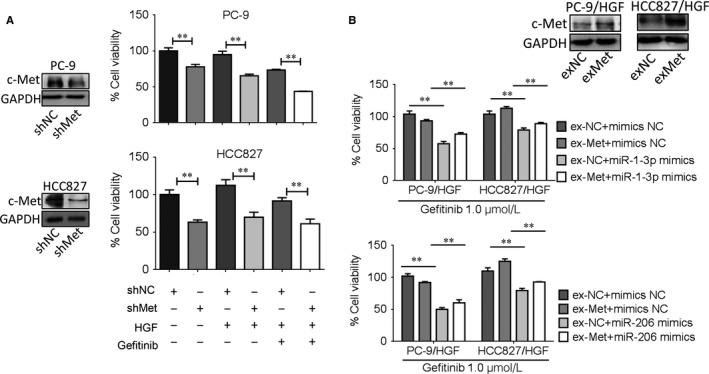
miR‐1‐3p/miR‐206 reversed HGF‐induced gefitinib resistance by c‐Met. A, c‐Met knockdown mimicked the effect of miR‐1‐3p and miR‐206. Left, c‐Met knockdown was evaluated by western blot assay in PC‐9 and HCC827 cells. Right, PC‐9 and HCC827 cells with c‐Met knockdown were treated with or without gefitinib (1 μmol/L) in the presence/absence of HGF(50 ng/mL) for 72 h, cell viability was determined by MTT assay. Data are means of three separated experiments ± SD, **P < .01. B, Top, c‐Met overexpression was evaluated by Western blot assay in PC‐9 and HCC827 cells. Bottom, c‐Met overexpression attenuated the effects of miR‐1‐3p and miR‐206. PC‐9/HGF and HCC827/HGF cells were co‐transfected with c‐Met overexpression plasmid and miR‐1‐3p/miR‐206 mimics and then treated with gefitinib (1 μmol/L), cell viability was determined after 48 h of treatment by MTT assay. Data are means of three separated experiments ± SD, **P < .01

To confirm the roles of c‐Met in overcoming HGF‐induced gefitinib resistance by miR‐1‐3p and miR‐206, we analysed whether c‐Met knockdown by shRNA can mimic the effect of miR‐1‐3p and miR‐206 transfection. The results are shown in Figure 4A, and the expression levels of c‐Met protein in c‐Met‐shRNA (sh‐Met) groups were significantly reduced compared with that in negative control shRNA (sh‐NC) groups. Furthermore, shRNA targeting c‐Met significantly sensitized HGF‐treated PC‐9 and HCC827 cells to gefitinib. The effects were similar to those of ectopic miR‐1‐3p/miR‐206 expression.

Next, we sought to examine whether c‐Met overexpression can attenuate the effects of miR‐1‐3p/miR‐206 on HGF‐induced gefitinib resistance. To this end, c‐Met was ectopically expressed in PC‐9 and HCC827 cells, respectively, which were confirmed by Western blot analyses (Figure 4B). Our results revealed that when c‐Met was ectopically expressed, there was a significant decrease of gefitinib sensitivity in PC‐9/HGF and HCC827/HGF cells with miR‐1‐3p/miR‐206 mimic transfection, indicating that re‐expression of c‐Met was capable of partially neutralizing the effect of miR‐1‐3p and miR‐206. Therefore, targeting c‐Met is involved in miR‐1‐3p and miR‐206 overcoming HGF‐induced gefitinib resistance.

### 3.5 miR‐1‐3p/miR‐206 suppresses c‐Met/Akt and Erk pathway in HGF‐induced gefitinib‐resistant cells

**Figure 5:**
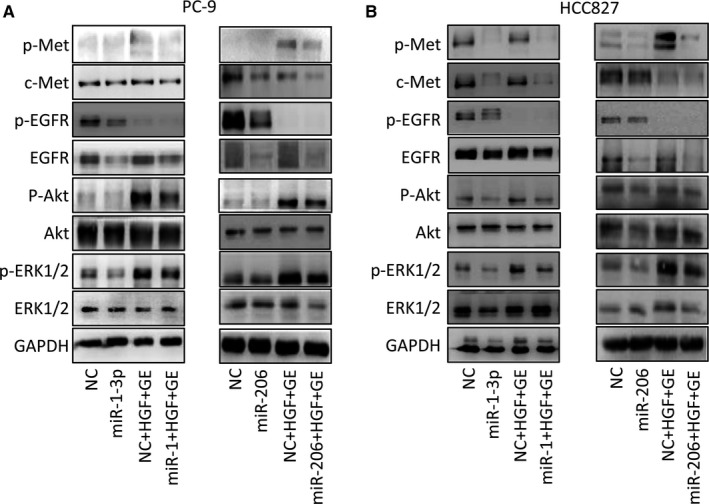
miR‐1‐3p/miR‐206 suppresses c‐Met/Akt and Erk pathway in HGF‐mediated gefitinib‐resistant cells. miR‐1‐3p and miR‐206 inhibited Akt and Erk1/2 signalling, even in HGF treated PC‐9 (A) and HCC827 (B) cells. PC‐9 and HCC827 cells were transfected with miR‐1‐3p or miR‐206 mimics and then treated with gefitinib (1 μmol/L) in the presence/absence of HGF (50 ng/mL) for 1 h, and cell extracts were prepared and immunoblotted with the indicated antibodies. GE: gefitinib

Some previous reports have showed that HGF induced gefitinib resistance through PI3K/Akt and Erk signalling pathway.^29^, ^30^, ^31^ To determine whether PI3K/Akt pathway is also an important mechanism in overcoming HGF‐induced gefitinib resistance by miR‐1‐3p/miR‐206, we examined the activities of c‐Met, EGFR, Akt and Erk by Western blotting. The results showed that miR‐1‐3p or miR‐206 alone decreased the expression of c‐Met in different degree (Figure 5A,B). Gefitinib alone can inhibit EGFR, Akt and Erk activities in PC‐9 and HCC‐827cells, but inhibitory effect is attenuated in presence of HGF (Figure S3). However, the combination of miR‐1‐3p or miR‐206 mimics and gefitinib, significantly inhibited HGF‐induced p‐c‐Met expression. Moreover, the combination of miR‐1‐3p or miR‐206 mimics and gefitinib also obviously inhibited phosphorylation of Akt and Erk1/2 in both cell lines. Interestingly, we also found that miR‐1‐3p or miR‐206 mimic transfection could suppress the EGFR expression in PC‐9 and HCC827 cells, which may increase the effect of two miRNAs on overcoming gefitinib resistance. Therefore, our results suggested that miR‐1‐3p/miR‐206 is able to down‐regulate c‐Met and EGFR expression and inhibit downstream Akt and Erk pathways in HGF‐induced gefitinib‐resistant NSCLC cells.

### 3.6 miR‐1‐3p/miR‐206 inhibits EMT in HGF‐induced gefitinib‐resistant cells

**Figure 6:**
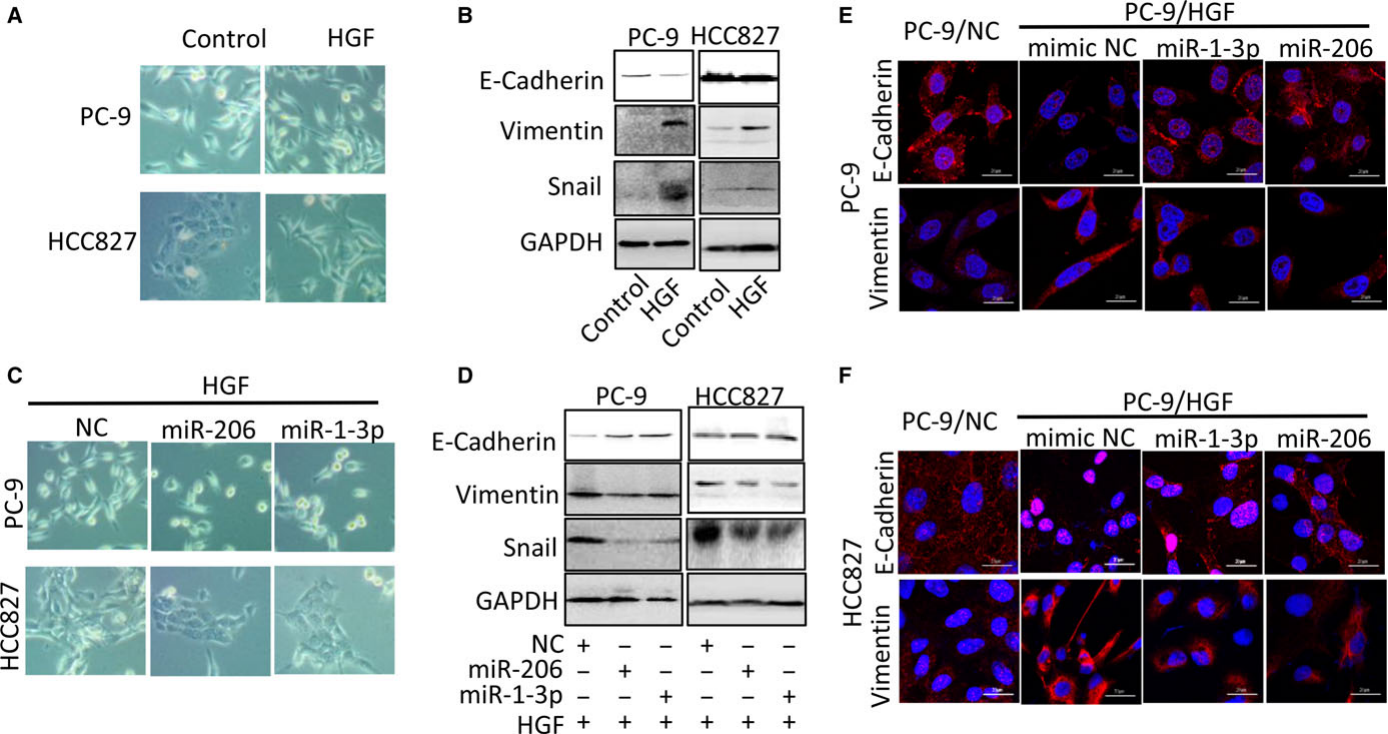
miR‐1‐3p/miR‐206 inhibits EMT in HGF‐mediated gefitinib‐resistant cells. A‐B, HGF induced‐transition from epithelial morphology to mesenchymal morphology (A) with increased EMT‐related molecular markers expression (B). PC‐9 and HCC827 cells were treated with HGF (50 ng/mL) for 48 h, the morphology was photoed by fluorescence microscope. EMT‐related molecular markers were detected by Western blot analysis. C‐D, miR‐1‐3p and miR‐206 mimics transfection inhibited HGF‐induced EMT. PC‐9 and HCC827 cells were transfected with miR‐1‐3p or miR‐206 mimics and then treated with HGF (50 ng/mL) for 48 h. The morphology was photoed by fluorescence microscope. EMT‐related molecular markers expressions were detected by Western blot analysis. E‐F, Immunofluorescence stain of EMT markers of E‐cadherin and Vimentin in PC‐9/NC, HCC827/NC, PC‐9/HGF and HCC827/HGF cells transfected with or without miR‐1‐3p/miR‐206 mimics. Scale bar: 20 μm

Epithelial‐mesenchymal transition (EMT) is extensively correlated with therapeutic resistance to EGFR‐TKIs,^32^, ^33^ and we next examined whether miR‐1‐3p and miR‐206 could reverse EMT in HGF‐induced gefitinib‐resistant cells. Typical epithelial morphology and expression of E‐cadherin, an epithelial marker, was observed in PC‐9 cells and HCC‐827 cells (Figure 6A,B), whereas mesenchymal morphology and high expression of Vimentin (a marker of mesenchymal phenotype) and Snail (a key regulator of EMT) were observed in HGF‐treated cells (Figure 6A,B). MiR‐1‐3p and miR‐206 treatment induced a transition from spindle‐like to epithelial‐like morphology (Figure 6C), as evidenced by the up‐regulation of E‐cadherin and down‐regulation of Vimentin and Snail in both HGF treated PC‐9 and HCC827 cells (Figure 6D). Immunofluorescence stain of EMT markers of E‐cadherin and Vimentin in PC‐9 cells and HCC‐827 cells confirmed that HGF‐induced EMT can be reversed by miR‐1‐3p and miR‐206 transfection (Figure 6E,F). These findings suggested that miR‐1‐3p and miR‐206 reverses EMT in HGF stimulated lung cancer cells might be another important mechanism correlated with overcoming resistance to gefitinib.

### 3.7 MiR‐1‐3p/miR‐206 overcame HGF‐induced gefitinib resistance in tumour xenografts

**Figure 7:**
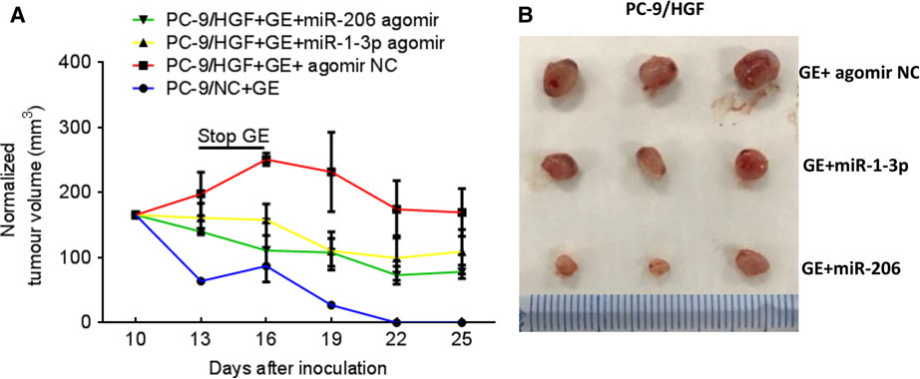
miR‐1‐3p/miR‐206 inhibits HGF‐mediated gefitinib resistance *in vivo*. A‐B, PC‐9/NC, and PC‐9/HGF cells (10^7^) were inoculated subcutaneously into nude mice on day 0. Mice received oral gefitinib (25 mg/kg/d) and/or locally injected miR‐1‐3p/miR‐206 agomirs, starting on day 10. The tumour size was measured every 3 days, and tumour volumes were calculated as described in Materials and Methods. We set three days of stop gefitinib (stop‐GE) interval to confirm the effect of gefitinib and combined treatment. Error bars indicate standard errors of 3 mice. B, macroscopic appearances of tumours harvested on day 25 are shown. GE:gefitinib

To further determine whether miR‐1‐3p and miR‐206 can overcome HGF‐induced gefitinib resistance *in vivo*, a gefitinib‐resistant xenograft tumour model (PC‐9/HGF) was used in the nude mice. MiR‐1‐3p and miR‐206 agomirs (more stable *in vivo* than mimics) were used to increase the expression of these two miRNAs. The results showed that PC‐9/NC tumours regressed rapidly in response to gefitinib treatment. Surprisingly, when we stopped gefitinib for 3 days (day14‐16), PC‐9/NC tumour grew again. Finally, PC‐9/NC tumours disappeared after 12 days of gefitinib treatment, whereas PC‐9/HGF tumours were slightly suppressed following gefitinib treatment (Figure 7A). Importantly, the combination of miR‐1‐3p (or miR‐206) and gefitinib reduced the size of PC‐9/HGF tumours (Figure 7A,B). Furthermore, MiR‐206+GE is more effective than MiR‐1‐3p+GE in our mouse models, which is consistent with the results *in vitro*. These results suggest that HGF can induce resistance to gefitinib *in vivo* and that this resistance can be overcome by miR‐1‐3p and miR‐206.

## 4 DISCUSSION

**Figure 8:**
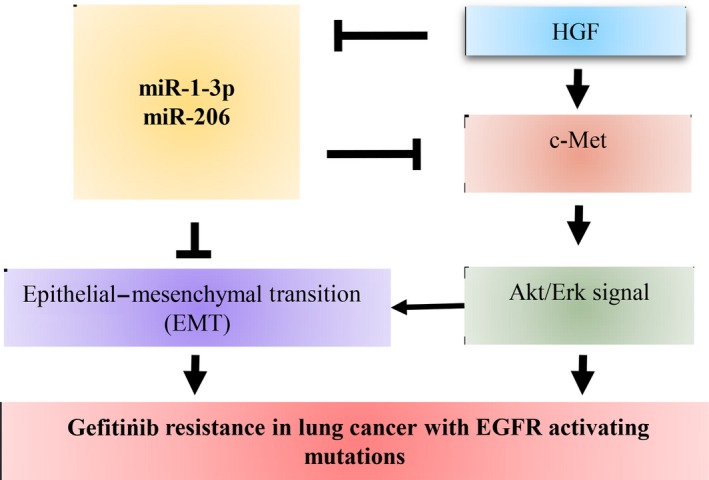
Proposed models on the inhibitory role of miR‐1‐3p/miR‐206 in HGF‐induced gefitinib resistance. As depicted in the model, miR‐1‐3p/miR‐206 overcome HGF‐induced gefitinib resistance by targeting c‐Met‐Akt/Erk pathway and epithelial‐mesenchymal transition (EMT) in lung cancer with EGFR activating mutation

Many miRNAs have been reported down‐regulated in lung cancer, such as miR‐34b/c,^7^ miR‐1,^25^ miR‐206,^17^ miR‐145^4^ and let 7.^5^ Among them, miR‐1‐3p and miR‐206, which belong to muscle‐specific miRNAs and play key roles in skeletal muscle differentiation, have found exhibited inhibitory function in lung cancer growth, migration and invasion in recent years.^16^, ^17^, ^18^ However, other functions of these two miRNAs are not known. In this study, we reported that miR‐1‐3p and miR‐206 can overcome HGF‐induced gefitinib resistance in EGFR mutant lung cancer cells *in vitro* and *in vivo*, and the mechanisms were related to inhibit Akt/Erk pathways and meanwhile suppress HGF‐induced EMT (Figure 8). The study showed the novel anti‐tumour function of miR‐1‐3p and miR‐206 in lung cancer.

MiR‐1‐3p and miR‐206 belong to the same miRNA cluster, which are frequently down‐regulated in various types of cancers. DNA methylation and acetylation of histones are two major epigenetic regulation mechanisms in miR‐1‐3p and miR‐206 expression; meanwhile, many nuclear transcription factors, such as NRF2,^17^ nuclear receptor subfamily 0, group B member 2, oestrogen‐related receptor γ, yin‐yang 1, and activator protein 1, were reported involved in miR‐206 regulation.^34^, ^35^ In this study, we showed that HGF induced a decrease in miR‐1‐3p/miR‐206 expression in both PC‐9 and HCC‐827 cells. In previous study, it has been reported that HGF stimulation enhanced Nrf2 activity in myotubes,^36^ and meanwhile another study revealed Nrf2 attenuate miR‐1 and miR‐206 expression by histone deacetylase 4 (HDAC4).^17^ Whether decreased miR‐1‐3p/miR‐206 expression by HGF depends on transcription factors Nrf2 and epigenetic regulation need further studies.

c‐Met play a key role in HGF‐induced gefitinib resistance. HGF overexpression‐induced TKIs resistance is associated with c‐Met phosphorylation and reactivation of phosphatidylinositol 3‐kinase (PI3K)/Akt signalling pathway in EGFR mutant NSCLC.^22^, ^30^ Targeting HGF‐c‐Met pathway by HGF antibody or c‐Met inhibitors restore the HGF‐induced resistance in lung cancer cells.^37^, ^38^ In addition, some miRNAs were also reported overcoming gefitinib resistance by targeting c‐Met in different types of lung cancer cells,such as in lung cancer stem cells (miR‐128),^39^ in primary or acquired gefitinib‐resistant lung cancer cells (mir‐19a, miR‐200a and miR‐130a),^40^, ^41^, ^42^ and in HGF‐induced lung cancer cells (miR‐34a).^43^ Consistent with these studies, we found that enforced expression of miR‐1‐3p/miR‐206 attenuated gefitinib resistance induced by HGF in EGFR mutant lung cancer cells, and the mechanisms were also involved in targeting c‐Met and its downstream pathways. To confirm the relationship of miR‐1‐3p/miR‐206 targeting c‐Met and HGF‐induced gefitinib resistance, we performed loss or gain of function assays. The results showed that c‐Met knockdown reduced the HGF‐induced gefitinib resistance. Instead, c‐Met overexpression increased HGF‐induced gefitinib resistance and attenuated the effect of miR‐1‐3p and miR‐206. From our results and others, we can find that c‐Met targeting is the common character in these studies, although the experiments were conducted under different gefitinib‐resistant conditions. Previous studies showed that miR‐206 target c‐Met directly in lung cancer cells.^44^ In our study, we further demonstrated that both miR‐1‐3p and miR‐206 have common target c‐Met in EGFR mutant PC‐9 and HCC‐827 cells. Furthermore, HGF‐c‐Met signalling induced gefitinib resistance with a decreased expression of miR‐1‐3p and miR‐206. Therefore, we suppose that combination of c‐Met inhibitors and c‐Met targeting miRNA, such as miR‐1‐3p/miR‐206, might be a better anti‐resistant strategy in overcoming HGF‐induced gefitinib resistance.

Epithelial‐mesenchymal transition is another mechanism of acquired EGFR‐TKI resistance in lung cancers. *In vitro* studies showed that the mesenchymal phenotype is more resistant to EGF‐TKI than the epithelial phenotype.^45^ Activated HGF/c‐Met pathway drives a mesenchymal phenotype in liver cancer has been reported.^46^ In our study, both morphologic observation and molecular marker detection by Western blot and immunofluorescence stain showed that HGF stimulation induced EMT in PC‐9 and HCC‐827 cells. We observed an elongated cell morphology, loss of E‐cadherin and increase in vimentin and snail expression. Whereas transfection of miR‐1‐3p and miR‐206 caused HGF‐expressed PC‐9 and HCC‐827 cells to undergo mesenchymal‐epithelial transition, the reverse of EMT. Together these findings indicate that suppressing EMT is another critical factor that miR‐1‐3p and miR‐206 overcoming HGF‐induced gefitinib resistance. Previous study reported that miR‐1 regulated EMT by directly target Slug gene in prostate cancer.^47^ However, whether EMT‐related genes are target directly by miR‐1‐3p and miR‐206 need further experimental verification.

In summary, we demonstrated *in vitro* and *in vivo* that miR‐1‐3p and miR‐206 can restore HGF‐induced gefitinib resistance in EGFR activating lung cancer cells. The effects are mediated by inhibition of Akt/Erk pathways and EMT.

## CONFLICTS OF INTEREST

The authors declare no conflict of interest.
