# Supplementary figures and images for: miR‐1‐3p and miR‐206 sensitizes HGF‐induced gefitinib‐resistant human lung cancer cells through inhibition of c‐Met signalling and EMT
Source: J Cell Mol Med. 2018 Apr 17;22(7):3526–36. doi: 10.1111/jcmm.13629 (PMC6010770; doi:10.1111/jcmm.13629)

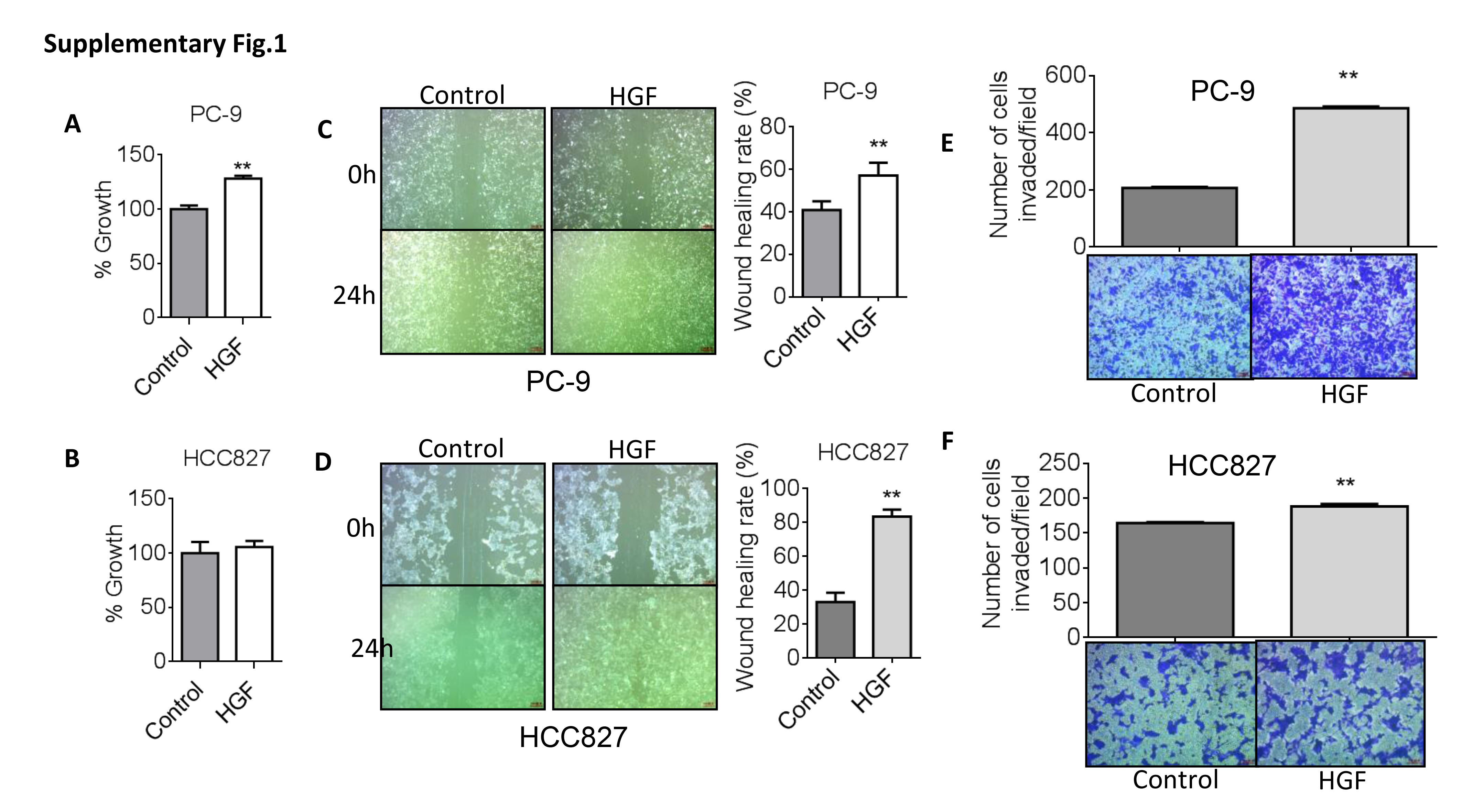

Supplement: Supplementary file 1 [file JCMM-22-3526-s001.tif]

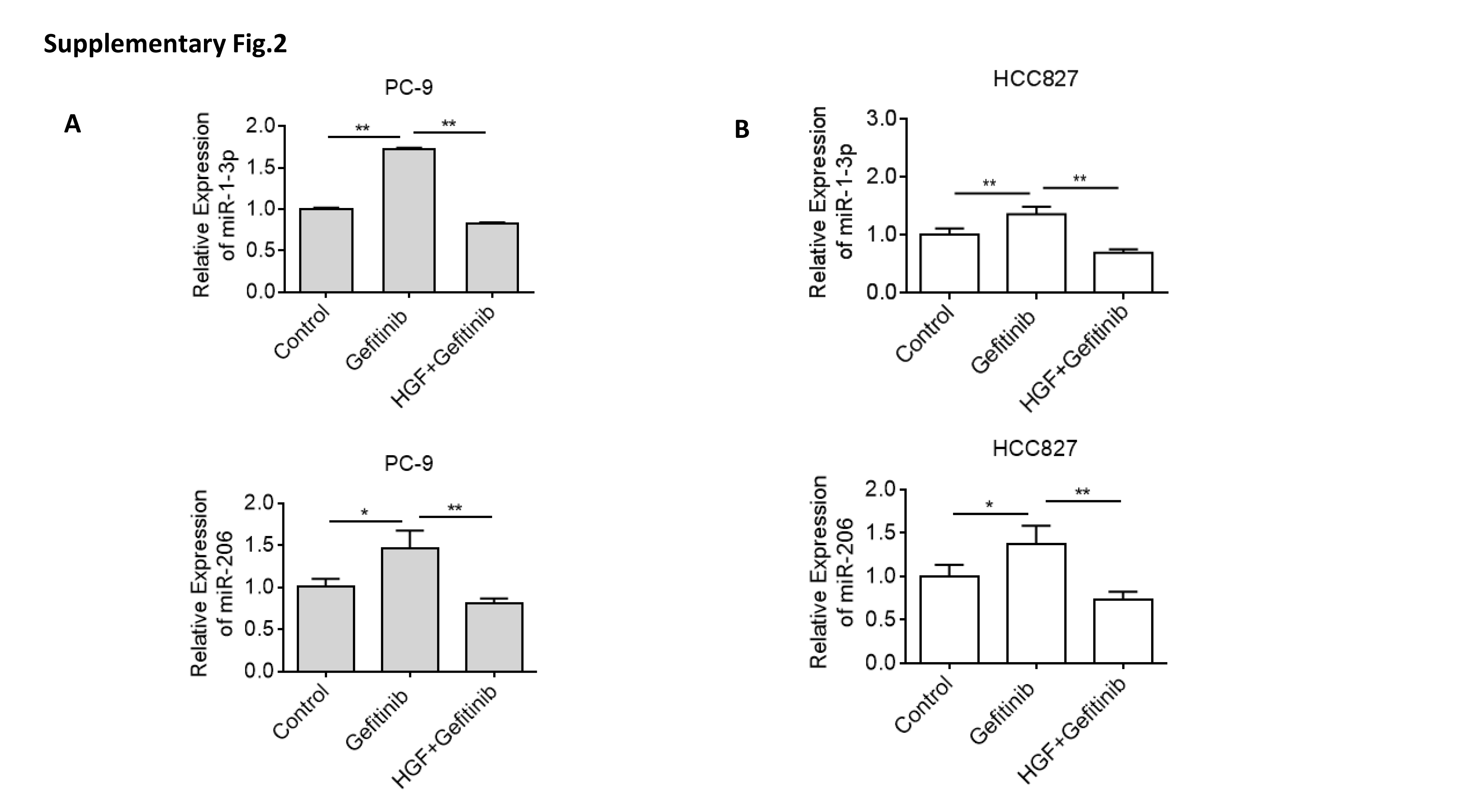

Supplement: Supplementary file 2 [file JCMM-22-3526-s002.tif]

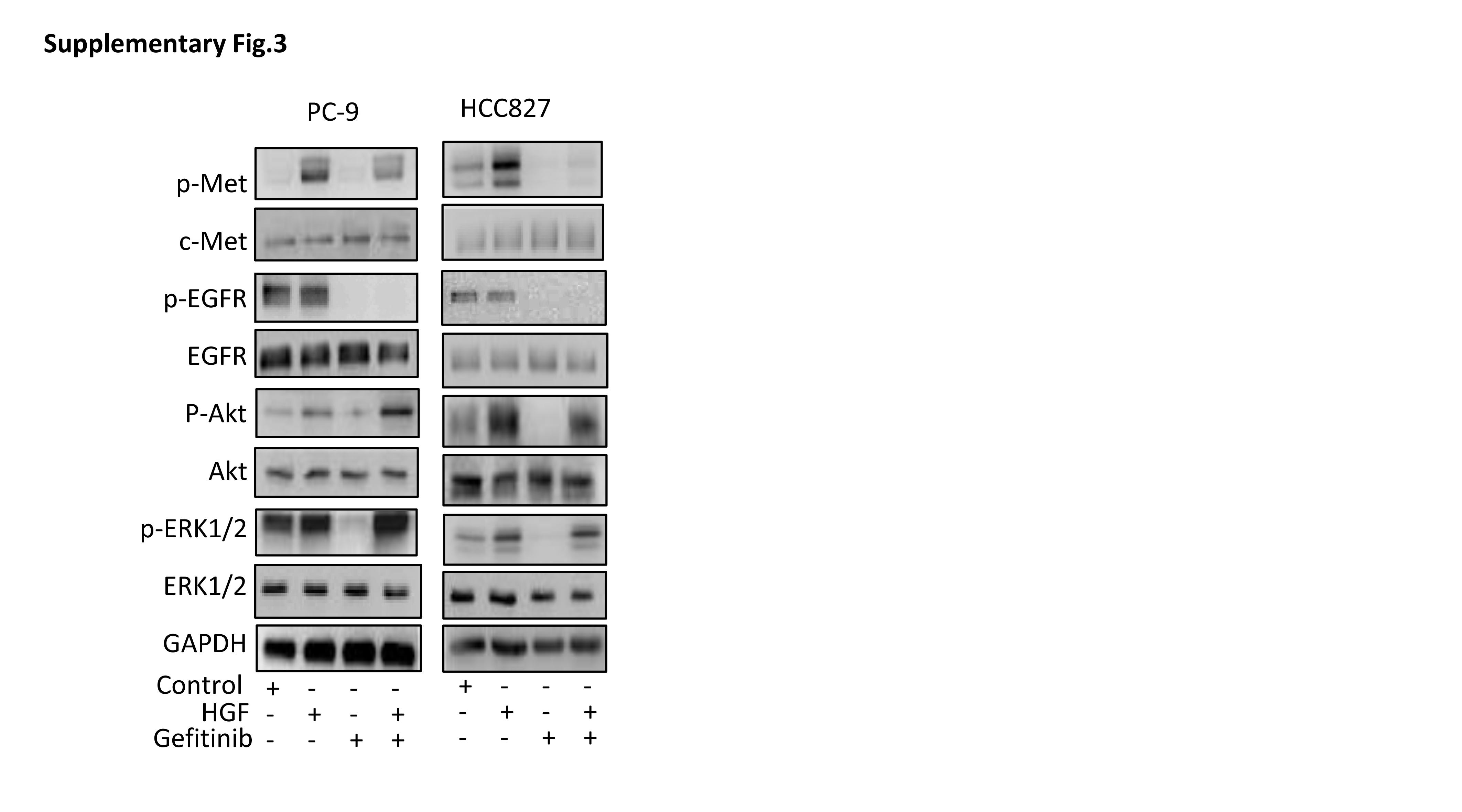

Supplement: Supplementary file 3 [file JCMM-22-3526-s003.tif]
