# Supplementary material for: miR‐1‐3p and miR‐206 sensitizes HGF‐induced gefitinib‐resistant human lung cancer cells through inhibition of c‐Met signalling and EMT
Source: J Cell Mol Med. 2018 Apr 17;22(7):3526–36. doi: 10.1111/jcmm.13629 (PMC6010770; doi:10.1111/jcmm.13629)
Supplement: Supplementary file 4 [file JCMM-22-3526-s004.doc]

**Supplementary table 1：**Sequences of miR-1-3p, miR-206 mimics and corresponding controls

| miRNAs | miR-1-3p Mimics | S: 5′- UGGAAUGUAAAGAAGUAUGUAU -3′；  A:5′- ACAUAC UUC UUUACAUUCCAUU -3′； |
| --- | --- | --- |
|  | miR-206 Mimics | S: 5′- UGGAAGUAAGGAAGUGUGUGG -3′；  A:5′- ACACACUUCCUUACAUUCCAUU -3′； |
|  | Mimics negative control | S:5′- UUCUCCGAACGUGUCACGUTT -3′；  A:5′- ACGUGACACGUUCGGAGAATT -3′； |
