# Supplementary material for: miR‐1‐3p and miR‐206 sensitizes HGF‐induced gefitinib‐resistant human lung cancer cells through inhibition of c‐Met signalling and EMT
Source: J Cell Mol Med. 2018 Apr 17;22(7):3526–36. doi: 10.1111/jcmm.13629 (PMC6010770; doi:10.1111/jcmm.13629)
Supplement: Supplementary file 5 [file JCMM-22-3526-s005.doc]

**Supplementary table 2：** synthesized MET shRNA and NC oligonucleotide insert

| **No** | **Sequence** |
| --- | --- |
| sh-MET1 | S: 5′- CACCGCAGTGAATTAGTTCGCTACGTTCAAGAGACGTAGCGAACTAATTCACTGCTT TTTTG-3′  A: 5′- GATCCAAAAAAGCAGTGAATTAGTTCGCTACGTCTCTTGAACGTAGCGAACTAATTC ACTGC-3′ |
| NC | S: 5′- CACCGTTCTCCGAACGTGTCACGTCAAGAGATTACGTGACACGTTCGGAGAATTTTTTG- 3′  A: 5′- GATCCAAAAAATTCTCCGAACGTGTCACGTAATCTCTTGACGTGACACGTTCGGAGA AC -3′ |
