# Supplementary material for: miR‐1‐3p and miR‐206 sensitizes HGF‐induced gefitinib‐resistant human lung cancer cells through inhibition of c‐Met signalling and EMT
Source: J Cell Mol Med. 2018 Apr 17;22(7):3526–36. doi: 10.1111/jcmm.13629 (PMC6010770; doi:10.1111/jcmm.13629)
Supplement: Supplementary file 6 [file JCMM-22-3526-s006.doc]

**Supplementary table 3：**The RT and PCR primers of miRNAs used in qRT–PCR analysis.

| miR-1-3p | RT primer | CTCAACTGGTGTCGTGGAGTCGGCAATTCAGTTGAGTACATACT |
| --- | --- | --- |
|  | PCR primer | F: ACACTCCAGGTGGGTGGAATGT |
|  |  | R: CTCAACTGGTGTCGTGGAG |
|  |  |  |
| miR-206 | RT primer | CTCAGCGGCTGTCGTGGACTGCGCGCTGCCGCTGAGCCACACAC |
|  | PCR primer | F: GGCGGTGGAATGTAAGGAAG |
|  |  | R: GGCTGTCGTGGACTGCG |
