# Supplementary material for: miR‐1‐3p and miR‐206 sensitizes HGF‐induced gefitinib‐resistant human lung cancer cells through inhibition of c‐Met signalling and EMT
Source: J Cell Mol Med. 2018 Apr 17;22(7):3526–36. doi: 10.1111/jcmm.13629 (PMC6010770; doi:10.1111/jcmm.13629)
Supplement: Supplementary file 7 [file JCMM-22-3526-s007.doc]

**Supplementary table 4：**The inserted sequence used in luciferase reporter constructs

| pmirGLO-c-Met-wt | 4741 gagcggcagc cccagaacag gccactcatt tagaattcta gtgtttcaaa acacttttgt  4801 gtgttgtatg gtcaataaca tttttcatta ctgatggtgt catt**cac**cca ttaggtaa**ac**  **4861 attcc**ctttt aaatgtttgt ttgttttttg agacaggatc tcactctgtt gccagggctg  4921 tagtgcagtg gtgtgatcat agctcactgc aacctccacc tcccaggctc aagcctcccg  4981 aatagctggg actacaggcg cacaccacca tccccggcta atttttgtat tttttgtaga  5041 gacggggttt tgccatgttg ccaaggctgg tttcaaactc ctggactcaa gaaatccacc  5101 cacctcagcc tcccaaagtg ctaggattac aggcatgagc cactgcgccc agcccttata  5161 aat**tttt**gta tagacattcc tttggttgga agaatattta taggcaatac agtcaaagtt  5221 tcaaaatagc atcacacaaa acatgtttat aaatgaacag gatgtaatgt acatagatga |
| --- | --- |
| pmirGLO–c-Met–mut | 4741 gagcggcagc cccagaacag gccactcatt tagaattcta gtgtttcaaa acacttttgt  4801 gtgttgtatg gtcaataaca tttttcatta ctgatggtgt cattgtgcca ttaggtaa**tg**  **4861** taaggctttt aaatgtttgt ttgttttttg agacaggatc tcactctgtt gccagggctg  4921 tagtgcagtg gtgtgatcat agctcactgc aacctccacc tcccaggctc aagcctcccg  4981 aatagctggg actacaggcg cacaccacca tccccggcta atttttgtat tttttgtaga  5041 gacggggttt tgccatgttg ccaaggctgg tttcaaactc ctggactcaa gaaatccacc  5101 cacctcagcc tcccaaagtg ctaggattac aggcatgagc cactgcgccc agcccttata  5161 aat**tata**gta tagtgtaagg tttggttgga agaatattta taggcaatac agtcaaagtt  5221 tcaaaatagc atcacacaaa acatgtttat aaatgaacag gatgtaatgt acatagatga |

Mutant sites were shown in red.
