# Supplementary material for: miR‐1‐3p and miR‐206 sensitizes HGF‐induced gefitinib‐resistant human lung cancer cells through inhibition of c‐Met signalling and EMT
Source: J Cell Mol Med. 2018 Apr 17;22(7):3526–36. doi: 10.1111/jcmm.13629 (PMC6010770; doi:10.1111/jcmm.13629)
Supplement: Supplementary file 8 [file JCMM-22-3526-s008.doc]

**Supplementary Fig.1 The effect of HGF on growth, migration and invasion of PC-9 and HCC827 cells. A-B.** HGF slightly promoted the growth of PC-9 cells **(A)**, but not in HCC827 cells **(B)**. Tumor cells were incubated with HGF (50ng/ml) for 72h and cell viability was determined by MTT assay. Data are means of three separated experiments ± SD, ***P* < 0.01 compared with gefitinib group. **C-D.** HGF promoted the migration of PC-9 cells **(C)** and HCC827 cells **(D)**. Tumor cells were incubated with HGF (50ng/ml). Left, representative wound healing assay images was taken before and after 24h of treatment. Right, would healing rate was also indicated. Data are means of three separated experiments ± SD, **P < 0.01. **E-F.** HGF promoted the invasion of PC-9 cells **(E)** and HCC827 cells **(F).** Tumor cells in the serum-free medium were plated in the upper chamber and induced invasion by HGF (50ng/ml) contained medium in the lower chamber. The number (Top) and representative images (Bottom) of the cells travelled through the membrane after 12h of treatment was shown. Data are means of three separated experiments ± SD, **P < 0.01.

**Supplementary Fig.2 Gefitinib up-regulated the expression of miR-1-3p and miR-206 in PC-9 and HCC827 cells, and HGF attenuated this effect in both cells. A-B.** Tumor cells were incubated with gefitinib (1μM) in presence/absence of HGF (50ng/ml) for 24h, the expression of miR-1-3p and miR-206 in PC-9(A) and HCC827 cells (B) were determined by QPCR assay. Data are means of three separated experiments ± SD, **P* < 0.05, **P < 0.01 compared with control group.

**Supplementary Fig.3 Gefitinib suppresses c-Met/Akt and Erk pathway but not in HGF-mediated PC-9 (A) and HCC827 (B) cells.** PC-9 and HCC827 cells were treated with gefitinib (1 μM) in presence/absence of HGF (50 ng/mL) for 1h, cell extracts were prepared and immunoblotted with the indicated antibodies.
